# The Neuromechanics of Inspiratory Muscles in Mechanical Ventilation Liberation Success and Failure

**DOI:** 10.7759/cureus.51570

**Published:** 2024-01-03

**Authors:** Hassan Aljohani, Derek Russell, Young-il Kim, John Bassler, John Lowman

**Affiliations:** 1 Respiratory Therapy Department, College of Applied Medical Sciences, King Saud Bin Abdulaziz University for Health Sciences, Riyadh, SAU; 2 Pulmonary, Allergy, and Critical Care Medicine, University of Alabama at Birmingham, Birmingham, USA; 3 Preventive Medicine, University of Alabama at Birmingham, Birmingham, USA; 4 Biostatistics, University of Alabama at Birmingham, Birmingham, USA; 5 Physical Therapy, University of Alabama at Birmingham, Birmingham, USA

**Keywords:** extra-diaphragmatic muscles, diaphragm, neuro-mechanical efficiency, mechanical ventilation, surface electromyography

## Abstract

Background: Assessing the neuromechanical coupling of inspiratory muscles during mechanical ventilation (MV) could reveal the physiological mechanism of MV failure. This study examined the respiratory neuromechanical characteristics between MV liberation success and failure.

Methods: This is an observational prospective study that included patients during their ventilator liberation process. Assessment of surface electromyography (sEMG) of inspiratory muscles, including the diaphragm and extra-diaphragmatic (scalene, sternocleidomastoid, and parasternal) muscles, was performed 15 minutes after the initiation of spontaneous breathing trials. Neuromechanical efficiency of the diaphragm (NME_Dia_) and extra-diaphragmatic muscles (NME_Extra_) were compared in patients who were successfully liberated from MV with those who failed MV liberation within 72 hours after extubation.

Results: A total of 45 patients were enrolled and 28 were female (67%). The sample median age was 63 (IQR 47, 69) years old. One-third of patients failed MV liberation within 72 hours of their spontaneous breathing trials (SBTs). NME_Dia_ was significantly lower in patients who failed MV liberation with a root mean square of (M 0.27), (IQR 0.21, 0.37) compared with (M 0.371), (IQR 0.3, 0.631) for the success group (p=0.0222). The area under the curve for NME_Dia_ was lower in the failure group (M 0.270), (IQR 0.160, 0.370) and (M 0.485), (IQR 0.280, 0.683) for the success group (p=0.024). However, NME_Extra_ was not statistically different between the two groups.

Conclusion: Reduced NME_Dia_ is a predictor of MV liberation failure. NME_Extra_ was not a major contributor to MV liberation outcomes. Further studies should assess the performance of inspiratory muscles NME indices to predict MV liberation outcomes.

## Introduction

Despite clinical assessment advancements, the prolonged mechanical ventilation (MV) liberation process is estimated to occur in up to one-fifth of the ventilated patients, increasing length of stay and risk of mortality [1-4]. Thus, avoiding MV weaning failure with careful clinical decision-making is a vital component of critical care outcomes [5-7].

Different pathologic states have been linked to producing an imbalance between respiratory muscle capacity and respiratory muscle load; including respiratory muscle weakness, impaired neuromuscular function, lung diseases, etc., [2,7-9]. The decline of diaphragmatic function may be due to diaphragmatic atrophy resulting from a longer duration of MV [10]. The neural drive of inspiratory muscles was identified as a clinical biomarker in MV liberation status and can be useful in determining the treatment response in patients with COPD [11-13]. Crural diaphragmatic activity decreases proportionally to the level of MV support [14]. Similarly, the activation of extra-diaphragmatic respiratory muscles (e.g., intercostal, scalene, and sternocleidomastoid muscles) is increased in an effort to maintain ventilatory balance during acute loaded breathing and MV liberation failure [15,16]. Increased extra-diaphragmatic muscle activation during MV liberation may not be a definite indicator of a fatiguing diaphragm; rather, it is related to its level of dysfunction [17-19].

Theoretically, the use of neuro-mechanical coupling indices is a logical choice to be used in practice to titrate MV support to match the patient's demand. When work of breathing (WOB) increases, the neuro-respiratory drive also increases in order to maintain optimal ventilatory balance [9,20,21]. Neuromechanical uncoupling results when increased neural output is not translated into adequate ventilatory output [19]. Ventilatory insufficiency raises the neuro-respiratory drive and electrical activity to inspiratory muscles to a higher degree, resulting in neuro-mechanical uncoupling [22]. This is also seen during MV liberation failure, where patients have higher neuro-respiratory drive to the diaphragm compared to those who were successfully weaned [17,23].

The objective of the current study was to describe the neuromechanical characteristics of diaphragmatic and extra-diaphragmatic muscles during MV liberation using surface electromyography (sEMG) of the same muscles. We hypothesized that the inspiratory muscles of patients who successfully liberated from MV would have low neuro-respiratory drive and high neuro-mechanical efficiency (NME) when compared to patients who failed MV liberation. The study also examines the applicability of using indices from sEMG of inspiratory muscles as a tool to investigate MV clinical outcomes.

## Materials and methods

Study design and settings

This physiologic study uses a prospective observational design. The study was approved by the local Institutional Review Board (IRB-30002180). Participants were recruited from the University of Alabama at Birmingham (UAB) Hospital’s Medical Intensive Care Unit (MICU) and the Cardiopulmonary Critical Care Unit (CPCC).

Participants

Adults who had received invasive MV for at least 24 hours and were ready to begin a spontaneous breathing trial (SBT) were included in this study. Exclusion criteria were patients: (1) unable to follow commands, (2) recovering from thoracic surgeries, (3) with an active neurologic condition (i.e., active head trauma, brain tumor, stroke, spinal cord injury, myasthenia gravis, multiple sclerosis, amyotrophic lateral sclerosis, Parkinson’s disease, seizures, and diagnosed or suspected brain death); (4) with cardiac or diaphragmatic pacemakers; (5) on whom it was difficult to identify neck muscles or rib cage landmarks due to excessive adipose tissue or anatomical deformities.

Protocol

During the SBTs, all subjects were ventilated using pressure support ventilation (PSV) mode on Puritan Bennett™ 980 Ventilators (Medtronic, Minneapolis, MN, USA) with pressure support of 5 cm H_2_O and a positive end-expiratory pressure of 5 cm H_2_O, as this is the standard setting used for SBTs at the study site. Therefore, variations in the level of MV support among our sample were eliminated. The success of MV liberation was defined in this study as any patient who is alive and not on invasive MV for at least 72 hours after extubation. MV liberation failure was defined as when the patient was deceased or reintubated within the same duration. While MV support level and EMG data collection window were standardized in all patients, the duration of PSV before extubation was postponed for seven hours with different PSV levels. Measurements were collected once for each patient. However, if the subject was not extubated within the same day, measurements were repeated on the next day and previous measurements were not included for analysis.

**Figure 1:**
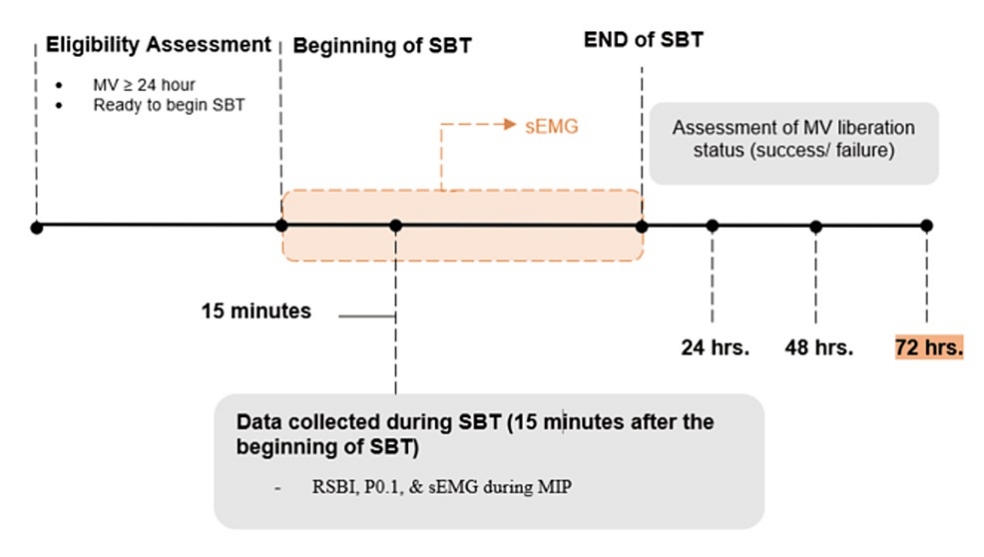
Study protocol and data collection timeline (MIP maximum inspiratory pressure; sEMG surface electromyography; P0.1 airway occlusion pressure; and RSBI rapid shallow breathing index). Image created by the authors.

Measurements were collected 15 minutes after the initiation of the SBT. The ventilatory measurements that were assessed included the patient’s maximum inspiratory pressure (MIP), rapid shallow breathing index (RSBI), and airway occlusion pressure (P0.1) (Figure 1). Measurements were collected during the respiratory therapist’s routine assessment using the available features on the MV, except for flow, which was measured using a spirometer (FE141 Spirometer, ADInstruments) and flow head (MLT1000L) attached to the inspiratory limb of the MV, proximal to the patient endotracheal tube. The flow measurement was used to synch inspiratory efforts with surface electromyography (sEMG) activity during MIP, which was measured during inspiratory occlusion, providing inspiratory pressure output.

EMG activity

The activity of diaphragmatic and extra-diaphragmatic muscles was evaluated by quantifying maximum EMG activity (EMGMAX%) and EMG area under the curve (EMGAUC%). EMG signals were collected at the initiation of the SBT and during MIP maneuvers. EMG activity was averaged across three measurements during SBT with a 3 to 5-minute delay to ensure the highest inspiratory effort.

The specific inspiratory muscles of interest were the diaphragm, scalene (SC), sternocleidomastoid (SCM), and parasternal intercostal muscles (para). sEMG electrodes were attached bilaterally to each muscle, and the best side of EMG activity was selected during the analysis. sEMG recordings for the diaphragm were placed over the 8th intercostal space between the mid and anterior axillary lines. EMG activity for the parasternal muscles was obtained from the 2nd intercostal space close to the sternum [24]. Sternocleidomastoid electrodes were placed at the lower part of the muscle belly, which was identified by submaximal neck flexion contraction. Scalene recordings were obtained from the posterior triangle of the neck between the SCM and the clavicles [25].

sEMG parameters were obtained using a portable electromyography system (Trigno™, Delsys). The sEMG signals were amplified and band-pass filtered between (40 Hz-3 kHz) with a sampling rate of 10 kHz [26,27]. The root mean square (RMS), an index of global EMG activity, was numerically calculated using fixed windows (duration = 1 milliseconds). The ratio of average sEMG activity during MIP to EMGMAX% and EMGAUC% was quantified. Inspiratory neuro-mechanical coupling parameters for each muscle were: (1) MIP/EMGMAX%, and (2) MIP/EMGAUC%.

Statistical methods

Statistical analysis was performed using SAS software, version 9.4 of the SAS System for Windows (SAS Institute Inc., Cary, NC, USA). Additional software used to complete the analysis and visualizations include R (R Core Team 2023, version ≥ 3.6.0, Vienna, Austria) which is freely available at http://www.R-project.org/. The assumptions of normality and homogeneity have been consistently violated according to Shapiro-Wilkes’s and Levene's tests. Results are therefore expressed as medians and interquartile ranges to measure central tendency and dispersion of data. Distribution was also measured, when appropriate, using frequency and percentage. For categorical outcomes, either the Chi-Square test of equal proportions or Fisher’s Exact test was implemented properly, based on sample size assumptions being met. For continuous variables, the Wilcoxon Rank Sum non-parametric test was performed for between-group comparisons. Missing data was sparse and considered missing at random (MAR) and, therefore, not included in descriptive measures or statistical tests. A control for bias was utilized by performing an analysis of EMG with blinding of the MV outcome (success vs. failure).

## Results

**Figure 2:**
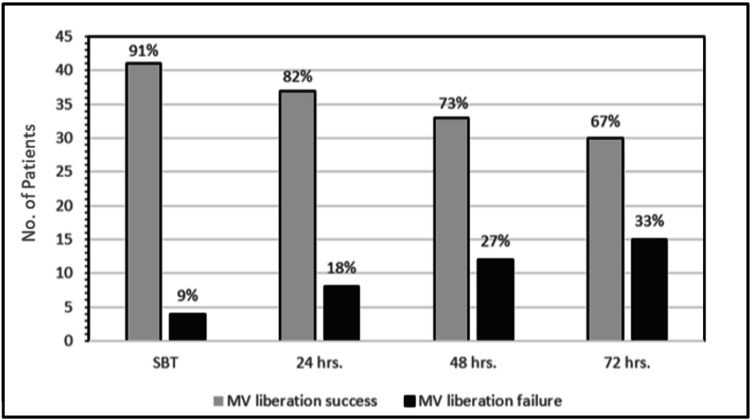
Numbers and percentages of mechanical ventilation success and failure across 72 hours after spontaneous breathing trial

A total of 45 patients who were receiving invasive MV support and were prepared for MV liberation and discontinuation were enrolled. Thirty (67%) patients were successfully liberated from MV support, and 15 (33%) patients failed MV liberation due to the occurrence of one of the following events within 72 hours of MV removal: failed liberation trail, the need for re-intubation or re-instating invasive MV support, or death (Figure 2).

Females accounted for 62% and males for 38% of the total sample. The median age was 67 (40, 79) years for the success group, and 58 (47, 68) years for the failure group. Body mass index for the patients ranged from normal weight to overweight and obese but was not significantly different across groups. Seventeen subjects (38% of the cohort) had chronic pulmonary disease. Median MV duration was 7.0 days (4.0, 12.0) and was significantly higher in the failure group compared to 3.0 days (2.0, 8.0) for the success group (p=0.0277). Patients with previous failed extubation attempts ranged from 13% to 17% in both groups. Occlusion pressure (P0.1) was not significantly different between the two groups. Three patients in the failure group and one in the success group were missing variables for BMI, and two in the failure and four in the success group were missing values for P_0.1_ (Table 1).

**Table 1:** Demographic and clinical baseline characteristics between MV liberation success and failure groups ^1^Chi-Square p-value; ^2^Wilcoxon rank sum p-value; ^3^Fisher Exact p-value, * Missing data by Failure/Success: BMI 3/1. BMI body mass index; COPD chronic pulmonary obstructive disease; ARDS acute respiratory distress syndrome; MV mechanical ventilation; IQR interquartile range.

	MV liberation Status			
	Failure (N=15)	Success (N=30)	Total (N=45)	
	Median (IQR)			P-value
Sex, n (%)				0.8279^1^
Female	9 (60%)	19 (63%)	28 (62%)	
Male	6 (40%)	11 (37%)	17 (38%)	
Age	67 (40, 79)	58 (47, 68)	63 (47, 69)	0.4999^2^
BMI *	28.3 (20.2, 32.5)	29.8 (25.1, 33.6)	29.5 (24.6, 33.6)	0.4649^2^
Pulmonary Disease, n (%)				0.2057^3^
Yes	7 (47%)	10 (33%)	17 (38%)	
Asthma	0 (0%)	3 (10%)	3 (7%)	
COPD	2 (13%)	4 (13%)	6 (13%)	
ARDS	5 (33%)	3 (10%)	8 (18%)	
Reason for admission, n (%)				0.7255^3^
Cancer	2 (13%)	4 (13%)	6 (13%)	
Cardiovascular	1 (7%)	1 (3%)	2 (4%)	
Infectious Disease	4 (27%)	2 (7%)	6 (13%)	
Liver	1 (7%)	5 (17%)	6 (13%)	
Neurological	2 (13%)	5 (17%)	7 (16%)	
Renal	2 (13%)	4 (13%)	6 (13%)	
Respiratory	2 (13%)	4 (13%)	6 (13%)	
Other	1 (7%)	5 (17%)	6 (13%)	
Days of MV	7.0 (4.0, 12.0)	3.0 (2.0, 8.0)	4.0 (2.0, 8.0)	0.0277^2^
Previous failed extubation, n (%)	2 (13%)	5 (17%)	7 (16%)	1.0000^3^

**Figure 3:**
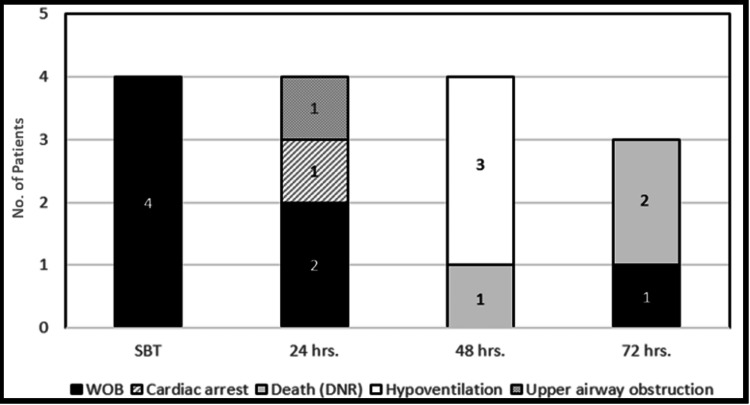
Reasons for mechanical ventilation liberation failure within a 72 hrs window from the spontaneous breathing trial

The number of patients with MV liberation failure doubled from 4 (9%) at SBT to eight (18%) in 24 hours and continued to rise at 48 hours to 12 patients (27%) until it reached 15 patients (33%) at 72 hours (Figure 2). The main reason for MV failure was increased work of breathing in seven of the MV liberation failure group (47%). Death in patients with do not resuscitate orders (n=3, 20%), hypoventilation (n=3, 20%), cardiac arrest (n=1, 7%), and upper airway obstruction (n=1, 7%) accounted for the rest of reasons of MV failure (Figure 3).

The failure group had a significantly lower MIP of 21 cm H_2_O compared to 32 cm H_2_O for the success group (p=0.0414). RSBI was significantly higher in the failure group versus the success group (95 and 60, respectively, p=0.0241) (Table 2). Neuromechanical efficiency (NME) of the diaphragm muscle was significantly lower for the failure group, with a median RMS of 0.270 (0.210, 0.370) and 0.371 (0.300, 0.631) for the success group (p=0.0222). Similarly, the NME of the diaphragm muscle was significantly lower for the failure group, with a median area under the curve (AUC) of 0.270 (0.160, 0.370) and 0.485 (0.280, 0.683) for the success group (p=0.024) (Table 3).

**Table 2:** Differences in MV liberation parameters between MV liberation success and failure groups ^1^Wilcoxon rank sum p-value, * Missing data by Failure/Success: P_0.1_ 2/4 MIP maximum inspiratory pressure; RSBI rapid shallow breathing index. * Missing data by Failure/Success: P_0.1_ 2/4

MV liberation status			
	Failure (N=15)	Success (N=30)	P-value
Median (IQR)			
MIP (cm H_2_0)	21 (35, 14)	32 (48, 23)	0.0414^1^
RSBI	95 (60, 107)	60 (44, 76)	0.0241^1^
P_0.1_ * (cm H_2_0)	-0.8 (-1.2, -0.4)	-1.1 (-2.1, -0.5)	0.0951^1^

**Table 3:** Neuromechanical characteristics of inspiratory muscles in MV liberation success and failure groups ^1^Wilcoxon rank sum p-value. MV Mechanical ventilation; RMS root mean square; AUC area under the curve; NME neuro-mechanical efficiency; Dia diaphragm, Extra extra-diaphragm.

	MV liberation status		
	Failure (N=15)	Success (N=30)	P-value
	Median (IQR)		
NME_Extra _(RMS)	0.276 (0.180, 0.690)	0.409 (0.255, 0.540)	0.3927^1^
NME_Dia _(RMS)	0.270 (0.210, 0.370)	0.371 (0.300, 0.631)	0.0222^1^
NME_Extra _(AUC)	0.270 (0.184, 0.822)	0.454 (0.247, 0.620)	0.3666^1^
NME_Dia _(AUC)	0.270 (0.160, 0.370)	0.485 (0.280, 0.683)	0.0243^1^

NME of extra-diaphragmatic muscles was not significantly different between the two groups for both RMS and AUC. However, extra-diaphragmatic muscles exhibited lower NME for the failure group, RMS 0.276 (0.180, 0.690) and AUC 0.270 (0.184, 0.822) compared to 0.409 (0.255, 0.540) and 0.454 (0.247, 0.620) for the success group (Tables 3, 4). Patients who failed because of high WOB exhibited higher diaphragmatic (p=0.032) and SCM (p= 0.046) activity for AUC% (Table 5).

**Table 4:** Differences in EMG activity of inspiratory muscles between MV liberation success and failure groups ^1^Wilcoxon rank sum p-value. MV Mechanical ventilation; RMS Root mean square; AUC area under the curve; Dia diaphragm; Extra extra-diaphragm; SC scalene; SCM sternocleidomastoid; para parasternal.

Table 4 Differences in EMG Activity of Inspiratory Muscles between MV Liberation Success and Failure Groups.			
	MV Liberation Success		
	Failure (N=15)	Success (N=30)	P-value
	Median (IQR)		
RMS Dia	100 (49.0, 100.0)	88 (45.0, 100.0)	0.397
AUC Dia	100 (58.0, 100.0)	74.0 (50.0, 100)	0.276^1^
RMS Extra	81 (55.3, 96.25)	79 (60, 98)	0.6383^1^
AUC Extra	67 (46.5, 95)	81 (67.5, 93)	0.6723^1^
RMS SCM	95 (69.5, 100)	95 (53, 100)	0.7819^1^
AUC SCM	100 (75, 100)	98 (68.5, 100)	0.6303^1^
RMS SC	94 (24, 100)	99 (67.25, 100)	0.3676^1^
AUC SC	100.00 (35.00, 100)	88.50 (67.50, 100)	0.9303^1^
RMS para	73.50 (44.25, 100)	86.00 (68.50, 100)	0.1783^1^
AUC para	87 (57.25, 100)	100 (83. 100)	0.3037^1^

**Table 5:** Comparison between patients Comparison between patients who failed MV liberation due to increased work of breathing and other patients who failed due to other reasons or who were successfully liberated. ^1^Wilcoxon rank sum p-value, * significant at 0.05. MIP maximum inspiratory pressure; RSBI rapid shallow breathing index; RMS root mean square; Dia diaphragm; Extra extra-diaphragm; SCM sternocleidomastoid; para parasternal; SC scalene; NME_Dia _neuromechanical efficiency of the diaphragm; NME_Extra _neuromechanical efficiency of the extra-diaphragmatic muscles; AUC area under the curve; WOB work of breathing.

	Failed due to WOB						
	No			Yes			
	M	(IQR)		M	(IQR)		P value
MIP	29	46	19	35	50	14	0.683
RSBI	67	47	92	107	49	122	0.097
RMS%							
Dia	85	45	100	100	86	100	0.052
SCM	91	69	100	100	71	100	0.151
SC	97	63	100	100	12	100	0.835
Para	100	78	100	84	58	100	0.538
Extra	90	74	99	92	61	100	0.605
NME_Dia _(cm H_2_O/µV)	0.358	0.612	0.271	0.350	0.500	0.146	0.364
NME_Extra_ (cm H_2_O/µV)	0.371	0.543	0.217	0.424	0.690	0.270	0.552
AUC%							
Dia	74	49	100	100	90	100	0.032*
SCM	98	68	100	100	100	100	0.046*
Sc	89	64	100	100	36	100	0.779
Para	82	66	100	100	39	100	0.841
Extra	86	69	96	89	70	100	0.47
NME_Dia_ (cm H2O/µV)	0.360	0.652	0.238	0.350	0.500	0.140	0.347
NME_Extra_ (cm H2O/µV)	0.363	0.625	0.207	0.370	0.822	0.270	0.707

## Discussion

The aim of this current study was to assess the neuromechanical characteristics of various inspiratory muscles in participants who were successfully liberated from MV as compared with those who did not. The results showed that one-third of the included patients required re-administration of full MV support following a spontaneous breathing trial. One of the novel findings of this study is that low diaphragmatic NME reflects an impaired ventilatory output and, therefore, is associated with poor MV liberation outcomes at 72 hours after SBTs. The study shows that the level of NME of extra-diaphragmatic muscles in our sample may not be a major predictor of MV status. The results of this study also provide evidence supporting the use of sEMG as an assessment tool for MV outcomes following SBTs.

Neuro-mechanical efficiency of the diaphragm

**Figure 4:**
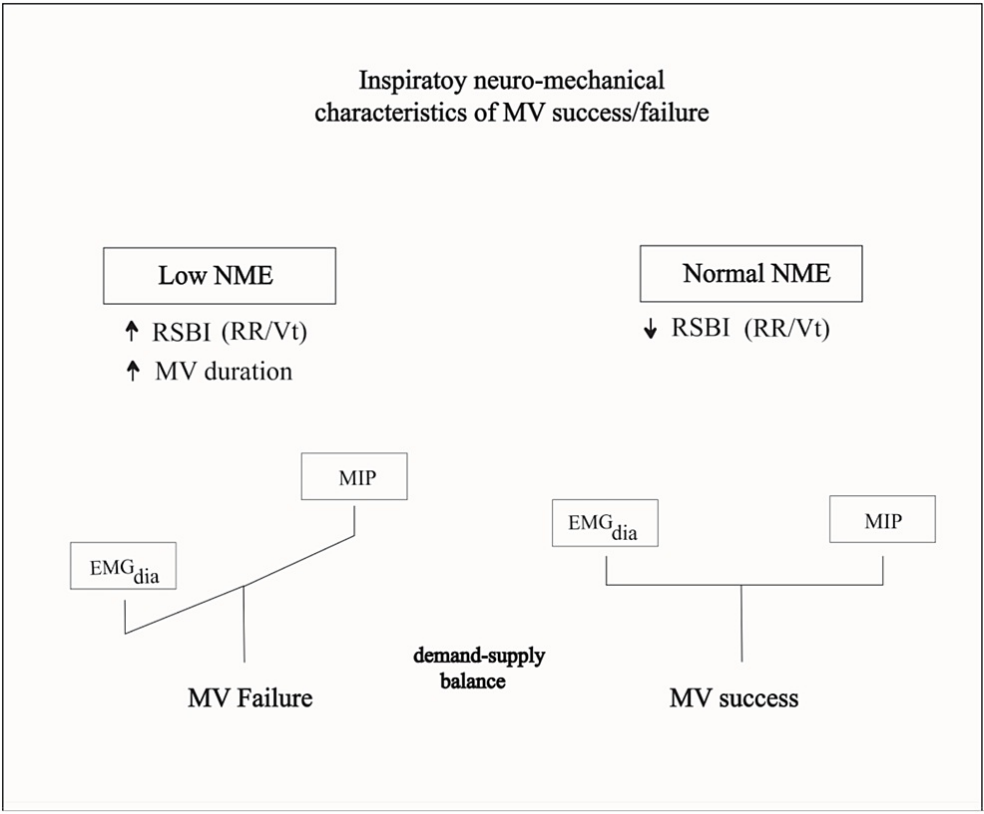
Theoretical framework for the characteristics of neuromechanical efficiency during MV liberation MV mechanical ventilation; NME neuro-mechanical efficiency; EMG_Dia_ Inspiratory electromyographic activity of the diaphragm muscles; MIP maximum inspiratory pressure; RSBI rapid shallow breathing index.

The failure group had an impaired diaphragmatic NME. This suggests that decreased diaphragmatic NME is a characteristic of patients who go on to fail MV liberation within 72 hours of their SBTs (Figure 4). The slightly higher diaphragmatic NRD in the failure group in our study demonstrates the value of assessing diaphragmatic NME to uncover the global picture of neuromechanical coupling as a predictor of MV liberation outcome.

Our results are consistent with other studies that highlight the inefficiency of the diaphragm to convert NRD into mechanical output [19,23]; this is characterized by higher RSBI and lower MIP in the failure group in our study. In another study that used continuous monitoring of the electrical activity of the diaphragm via an invasive esophageal catheter (Edi), the NME of the diaphragm was lower for the liberation failure group compared to the success group during SBT trials [28]. Diaphragmatic NME did not change over time regardless of MV outcomes, using Edi catheter and end-expiratory occlusion to calculate NME [29]. However, the level of MV support was not fixed among patients, which might explain the unchanged NME based on ventilatory demand.

Using similar measurement techniques, Bellani and colleagues, in a retrospective study, found that NME of the diaphragm was not linked to any of the clinical outcomes [29]. Yet, in contrast to our study, MV days and inspiratory muscle capacity reflected by parameters such as MIP were not different between high and low NME groups. Reduced NME of the diaphragm in the failure group in our cohort might be related to two major factors. Firstly, the high incidence of developing diaphragmatic weakness among critically ill patients and the reduced diaphragmatic contractility [30-34]. In our study, patients in the failure group had longer durations of MV, which reinforces the impact of MV on diaphragm weakness demonstrated in other studies [35]. Diaphragmatic dysfunction may result from structural changes and proteolysis, decreasing the cross-sectional area of diaphragm muscle fibers [31,36]. Secondly, although lung volumes were not assessed in this study, we cannot exclude the presence of intrinsic positive end-expiratory pressure (iPEEP), which could cause flattening of the diaphragm, placing it at a position that reduces its contractile capacity [37]. The high RSBI in the failure group reflects worsened pulmonary mechanics, which is also consistent with the results of Jubran and Tobin as a reason for failed MV liberation [16].

Neuro-mechanical efficiency of extra-diaphragmatic muscles

In contrast to our hypothesis, although NME of extra-diaphragmatic muscles tended to be lower for the failure group, it was not statistically different than the success group. This finding suggests that NME of extra-diaphragmatic muscles is unlikely to be a differential predictor of MV status among patients ventilated for an average of four days. A plausible explanation could be that measuring NME of extra-diaphragmatic muscles is not sensitive enough to MV outcome at 72 hours. However, our data revealed that patients who failed their MV liberation trial due to increased work of breathing had a significantly increased EMGAUC% activity for the diaphragm and SCM. Schmidt and colleagues found that neuromechanical coupling of extra-diaphragmatic muscles (EMG/tidal volume) was increased during low MV support compared to high-pressure support [21].

Using sEMG as an assessment tool of neuro-mechanics

To the best of our knowledge, most of the physiological studies that examined sEMG of extra-diaphragmatic muscles looked at simultaneous clinical responses during MV. In contrast, this study assessed MV liberation over a period of 72 hours using sEMG of respiratory extra-diaphragmatic muscles. A handful of studies have demonstrated sEMG as a valid tool for assessing respiratory mechanics and sensation during MV, with a diagnostic accuracy characterized as a high and unclear risk of bias [38]. In a systematic review, previous studies used sEMG as a tool to reflect the loading/unloading of respiratory muscles or respiratory sensation during changes in MV support [38]. Yet, all patients in our study received a consistent level of MV support (5 cm H_2_O) and PEEP (5 cm H_2_O) during their SBT.

Our study lays the groundwork for future research on various aspects of MV liberation. We propose that sEMG of inspiratory muscles can be used to categorize patients according to their MV liberation status. We suggest diaphragmatic NME as a bedside clinical tool for risk prediction and prognostication in patients being evaluated for ventilator liberation. Future studies should evaluate the performance of NME against other gold standard tests (e.g., RSBI) in predicting MV liberation outcomes. Moreover, the impact of prolonged MV on neuromechanical indices warrants further investigation.

Limitation

General limitations of sEMG could be considered a limitation due to adjacent muscle crosstalk, noise contamination, and difficulty finding optimal sensor positions [39]. Also, we cannot exclude the contribution of abdominal muscles in inspiratory pressure generation or contamination from adipose tissue, especially in patients with high BMI. Although we paid particular attention to excluding patients on sedatives, there was a non-significant trend toward a difference in P_0.1_. Thus, inspiratory effort might be variable between the two groups which could have biased the results because of prolonged sedation effect or other neurological disorders. Given its observational nature, the results of our study should be interpreted with caution due to the lack of a controlled environment.

## Conclusions

Our data showed that the neuromechanical coupling of the diaphragm decreases in patients who failed MV liberation within 72 hours. Extra-diaphragmatic muscle efficiency does not distinguish between patients destined to succeed or fail an MV liberation status attempt in our cohort. Moreover, the duration of MV predicts reduced diaphragmatic neuromechanical coupling and, therefore, MV liberation failure. These results were examined using sEMG of inspiratory muscles, which provides further evidence of its usefulness for assessing respiratory mechanics during MV in physiological studies.
